# Short-chain fatty acid valerate reduces voluntary alcohol intake in male mice

**DOI:** 10.1186/s40168-024-01829-6

**Published:** 2024-06-17

**Authors:** Suresh C. Bokoliya, Jordan Russell, Yair Dorsett, Hunter A. Panier, Vijender Singh, Lauren Daddi, Hanshu Yuan, Liv R. Dedon, Zhongmao Liu, Yuqi Zhou, Zefang Min, Jessica R. Barson, Jonathan Covault, Jason A. Bubier, Yanjiao Zhou

**Affiliations:** 1 Department of Medicine, University of Connecticut Health Center, Farmington, CT 06030 USA; 2 Computational Biology Core, University of Connecticut Health Center, Farmington, CT 06030 USA; 3 Calhoun Cardiology Center, University of Connecticut Health Center, Farmington, CT 06030 USA; 4 Department of Statistics, University of Connecticut, Storrs, CT 06269 USA; 5 Department of Neurobiology & Anatomy, Drexel University College of Medicine, Philadelphia, PA 19129 USA; 6 Department of Psychiatry, University of Connecticut Health Center, Farmington, CT 06030 USA; 7 The Jackson Laboratory, Bar Harbor, ME 04609 USA

**Keywords:** Alcohol drinking, SCFA, Sodium valerate, GABA, Microbiome

## Abstract

**Background:**

Despite serious health and social consequences, effective intervention strategies for habitual alcohol binge drinking are lacking. The development of novel therapeutic and preventative approaches is highly desirable. Accumulating evidence in the past several years has established associations between the gut microbiome and microbial metabolites with drinking behavior, but druggable targets and their underlying mechanism of action are understudied.

**Results:**

Here, using a drink-in-the-dark mouse model, we identified a microbiome metabolite-based novel treatment (sodium valerate) that can reduce excessive alcohol drinking. Sodium valerate is a sodium salt of valeric acid short-chain fatty acid with a similar structure as γ-aminobutyric acid (GABA). Ten days of oral sodium valerate supplementation attenuates excessive alcohol drinking by 40%, reduces blood ethanol concentration by 53%, and improves anxiety-like or approach-avoidance behavior in male mice, without affecting overall food and water intake. Mechanistically, sodium valerate supplementation increases GABA levels across stool, blood, and amygdala. It also significantly increases H4 acetylation in the amygdala of mice. Transcriptomics analysis of the amygdala revealed that sodium valerate supplementation led to changes in gene expression associated with functional pathways including potassium voltage-gated channels, inflammation, glutamate degradation, L-DOPA degradation, and psychological behaviors. 16S microbiome profiling showed that sodium valerate supplementation shifts the gut microbiome composition and decreases microbiome-derived neuroactive compounds through GABA degradation in the gut microbiome.

**Conclusion:**

Our findings suggest that sodium valerate holds promise as an innovative therapeutic avenue for the reduction of habitual binge drinking, potentially through multifaceted mechanisms.

Video Abstract

**Supplementary Information:**

The online version contains supplementary material available at 10.1186/s40168-024-01829-6.

## Background

Excessive alcohol consumption poses a significant public health concern, leading to a myriad of health and social problems as well as a substantial economic burden [1, 2]. Alcohol use disorder (AUD), which impacts nearly 29.5 million individuals aged 12 and older in the USA [3, 4], frequently co-occurs with anxiety disorders [5]. Drugs like acamprosate, disulfiram, and naltrexone have received approval from the US FDA for treating AUD [6, 7]. Concurrently, several promising candidates, including ketamine [8], are presently under investigation. Nonetheless, the US FDA has not approved any new drugs for AUD treatment in the past two decades. The development of pharmacological interventions to reduce alcohol drinking remains a high priority for the mission of the National Institute on Alcohol Abuse and Alcoholism [9]. Binge drinking, excessive alcohol consumption in shorter periods of time, is the most common and costly pattern of excessive alcohol use in the USA [2, 10]. Studies indicate that one out of three young Europeans and North Americans regularly engage in binge drinking [11]. Binge drinking has been linked to a heightened risk of developing AUD and neuropsychiatric disorders in later life [12, 13].

Recent advances in microbiome and alcohol research have shown that AUD negatively affects not only the liver and other organ systems but also the gastrointestinal system and its indigenous microbiota. Individuals with AUD exhibit alterations in their gut microbiome, characterized by reduced levels of bacteria associated with anti-inflammatory effects and the production of short-chain fatty acids (SCFAs)-the primary gut microbial metabolites derived from the fermentation of non-digestible carbohydrates [14, 15]. Emerging evidence on binge drinking suggests that it leads to significant alteration in the microbiome, with some effects persisting from adolescence into adulthood of rats [16]. These alterations may further accelerate the cycle of addiction via the gut-brain axis [17]. Interestingly, the gut microbiome is closely linked to SCFA production and alcohol consumption directly decreases the abundance of SCFA-producing bacteria [14, 18], and lowers SCFA levels in stools and blood [19, 20]. SCFAs are vital in regulating immune responses and maintaining gut and blood–brain barrier integrity [21]. SCFAs have also been shown to affect behavior, including those involved in reward, stress [22], and substance use disorders [23]. In rats, administration of SCFAs, like sodium butyrate, reduced alcohol-induced liver damage and inflammation [24]. Systemic use of sodium butyrate even produced antidepressant-like behavior in rats [25]. In contrast, acetate, another SCFA, encourages ongoing heavy drinking [26]. At the molecular level, modulation of epigenetic regulation and gene expression in the brain, potentially mediated by SCFAs, may contribute to their effects on alcohol-drinking behavior [27, 28].

GABA is reported to play a crucial role in neuropsychiatric, neurological disorders, and AUD [29–31]. Several studies reported lower GABA levels in abstinent individuals with AUD, particularly in the occipital cortex [30] and anterior cingulate cortex [32]. Investigations focusing on GABA have also noted decreased levels in binge drinkers compared to light drinkers in the anterior cingulate cortex [33]. Given its structural similarities with GABA [34], valeric acid, an SCFA, was a central focus of our study for its potential involvement in compensatory neurochemical adjustments linked to binge drinking.

The main aim of this study was to evaluate alcohol consumption in a mouse model of binge drinking following supplementation with valeric acid. Additionally, we investigated the impact of valeric acid on the gut microbiome, anxiety-like behavior, and depression. Furthermore, we aimed to elucidate several potential mechanisms of action of valeric acid, including its effects on epigenetic modulation and regulation of GABA.

## Methods

### Animals

Male C57BL/6 J mice, (RRID: IMSR_JAX:000664), aged 6–8 weeks, were obtained from Jackson Laboratories (ME, USA) for the study. The mice were housed individually under a reversed 12-h light/dark cycle, with lights off from 7:00 pm to 7:00 am. A minimum acclimation period of 2 weeks was provided for all animals before they were randomly assigned to their respective groups. Mice had free access to food (irradiated chow, Teklad Global 18% protein rodent diet, 2918, Inotiv, WI, USA) and deionized water to acclimate to the testing environment. Body weight and daily food and fluid intake were monitored and noted for all animal experiments.

### Antibiotics treatment

An antibiotic cocktail (Abx) comprised of 3.0 mg/mL vancomycin (Thermo Fisher Scientific, CA, USA), 6.0 mg/mL metronidazole (Sigma, MO, USA), 6.0 mg/mL ampicillin (Sigma, MO, USA), and 6.0 mg/mL neomycin (Janssen Pharmaceuticalaan, Belgium) was given to mice via oral-gastric gavage for five consecutive days (250 µL Abx/gavage/mouse). In contrast, control mice were given phosphate-buffered saline (PBS; Thermo Fisher Scientific, CA, USA) as control via oral-gastric gavage for five consecutive days, with each mouse receiving 250 µL of PBS per gavage.

### DID paradigm for binge-like ethanol drinking and SCFA administration

A standard 4-day drinking in the dark (DID) model was used to evaluate binge-like ethanol drinking patterns in mice. A 20% v/v ethanol solution was prepared by diluting 190-proof ethyl alcohol (Sigma, MO, USA) with deionized water. During days 1–3, the water bottles were temporarily removed during the dark cycle of the mice, and the mice were provided with a tube containing the 20% v/v ethanol solution for a duration of 2 h. On day 4, the assessment of binge-like ethanol consumption took place, where the mice were given access to the 20% v/v ethanol solution for a period of 4 h. The volume of fluid in the tube (read to the closest 100 µL) was measured immediately upon placement and at 2 h and 4 h into the test. Following the completion of the 4 h test, approximately 100 µL of blood was collected through cardiac puncture after CO_2_ euthanasia. Blood samples were subjected to centrifugation at 10,000 rpm for 10 min. The determination of blood ethanol concentrations (BEC) was performed using an Analox AM1 Analyzer (Analox Instruments, MA, USA). The quantity of ethanol consumed was then calculated as grams per kilogram of the mouse’s body weight.

All SCFA were used in sodium salt form. Briefly, sodium acetate (Sigma, MO, USA), sodium butyrate (Sigma, MO, USA), sodium valerate (Ambeed, IL, USA), and sodium chloride (NaCl; Sigma, MO, USA) were prepared at 200 mM concentration freshly in drinking water every 2 days. These concentrations were chosen based on earlier studies conducted in mice [35, 36]. All SCFA and NaCl solutions were pH verified to control for acidity effects on daily consumption. SCFAs were given to mice via oral drinking for 10 consecutive days before any experiment, and this regimen was maintained throughout the entire experiment.

### SCFA measurement

The fecal samples (50–100 mg) were mixed with 1 mL of a 0.5% phosphoric acid solution (Sigma, MO, USA). After collection, the samples were immediately frozen at − 20 °C. Upon thawing, the fecal content suspensions were thoroughly homogenized by vortexing for 2 min and then centrifuged at 18,000 × *g* for 10 min. The resulting supernatant was extracted with ethyl acetate (Sigma, MO, USA) and then centrifuged in glass tubes at 3000 × *g* for 10 min. The separated organic phase was analyzed using gas chromatography with a flame ionization detector (Shimadzu GC-QP2010 SE, Tokyo, Japan). Identification of SCFAs was achieved by comparison to the chemical standards (acetic acid, propionate acid, butyric acid, isovaleric acid, and valeric acid) (Sigma, MO, USA).

### Open field activity test

The open-field activity test was conducted to assess the impact of sodium valerate supplementation on exploratory and anxiety-like behavior. This test is a widely used method for analyzing locomotor ability and anxiety-related emotional behaviors in C57BL/6 J mice. The open field activity chamber, constructed from white Plexiglass and illuminated with daylight, had dimensions of 37.5 cm height × 40 cm length × 40 cm width. General locomotor activity was recorded for 10 min by an overhead camera and analyzed using an automated video tracking system (ANY-maze v.4.6, Stoelting, IL, USA, RRID:SCR_014289). The primary variables of interest included the total distance traveled (cm), the number of entries in the center area (20 cm length × 20 cm width), and the percentage of time spent in the center area of the open field.

### Measurement of GABA

GABA levels in plasma, stool, and amygdala samples were determined using an ELISA kit (LDN, Labor Diagnostika Nord, Nordhorn, Germany) following the manufacturer’s instructions. Plasma samples were used directly without any additional preparation. For stool and amygdala, samples, they were first thawed and then homogenized in a solution consisting of 0.01 N HCl (Thermo Fisher Scientific, MA, USA), 1 mM EDTA (Thermo Fisher Scientific, MA, USA), and 4 mM sodium metabisulfite (Thermo Fisher Scientific, MA, USA). After homogenization, the samples underwent centrifugation at 3000 × *g* for 5 min at 4 °C before GABA estimation. To summarize the assay procedure, plasma, homogenized stool, amygdala samples, and the kit’s standards were all processed on an extraction plate. They were subsequently derivatized using an equalizing reagent and subjected to a standard competitive ELISA conducted in GABA-coated 96-well microtiter strips. The optical density (OD) of the solution within the wells was rapidly read at 450 nm using a 96-well plate reader (iMark™ Microplate Absorbance Reader; Biorad, CA, USA, RRID:SCR_023799). The OD data was employed to determine the GABA concentration via a standard curve.

### Measurement of histone acetylation in amygdala

Amygdala samples were thawed, and the histone fractions were prepared using a commercial histone extraction kit (Abcam, Cambridge, UK) following manufacturers’ instructions. The bulk acetylation of histone H3 and H4 was determined using commercial ELISA kits according to the manufacturer’s instructions (Abcam, Cambridge, UK). The OD of the samples was measured using a 96-well plate reader at 450 nm (iMark™ Microplate Absorbance Reader; Biorad, CA, USA). The amounts of acetyl histone H3 and H4 were measured by comparing to the kit-supplied standards.

### RNASeq of the amygdala and data analysis

Total RNA was isolated from amygdala samples using the Direct-zol RNA Microprep Kit (Zymoresearch, CA, USA). Quantification of total RNA and assessment of its purity ratios were performed using the NanoDrop 2000 spectrophotometer (Thermo Fisher Scientific, MA, USA, RRID:SCR_018042).

Total RNA libraries were prepared for transcriptome sequencing using the Zymo-Seq RiboFree Stranded Total RNA library preparation kit (Zymo Research, CA, USA) following the manufacturer’s instructions. Further, rRNAs were depleted as part of the sample preparation process. Libraries were validated for length and adapter dimer removal using the Agilent TapeStation 4200 D1000 High Sensitivity assay (Agilent Technologies, CA, USA, RRID:SCR_019398). Subsequent quantification and normalization were carried out using the dsDNA High Sensitivity Assay for Qubit 3.0 (Life Technologies, CA, USA, RRID:SCR_020311). Sample libraries were then prepared for Illumina sequencing following the manufacturer’s protocol (Illumina, CA, USA). All the individual samples were consolidated into a single sequencing pool, with proportions targeting 30 M reads/sample, and sequenced on the Illumina NovaSeq 6000 platform (Illumina, CA, USA, RRID:SCR_016387).

Raw reads were subjected to adaptor removal and quality control including filtering of low-quality reads. Filtered reads were mapped to the mouse reference genome using STAR (Spliced Transcripts Alignment to a Reference, RRID:SCR_004463). Gene expression level was estimated by transcripts per million of transcript sequences. Principal component analysis (PCA) was performed to visualize overall gene expression differences between compared groups in several major principal components. The analysis of differential gene expression (DEG) was performed using the DESeq2 (RRID:SCR_015687) package for RNA-Seq data. The identified significantly regulated genes were displayed with varying adjusted *p* value thresholds. The Benjamini–Hochberg procedure also referred to as the false discovery rate (FDR) method, was applied to correct *p* values for multiple testing. We utilized Ingenuity Pathway Analysis (IPA, RRID:SCR_008653) to predict pathways associated with differentially expressed genes and employed Gene Set Enrichment Analysis (GSEA, RRID:SCR_003199) to visualize the outcomes in a heatmap. Right-tailed Fisher’s exact test was performed in the IPA to identify significant canonical pathways [37].

### 16S rRNA sequencing and microbiome data analysis

Microbial genomic DNA extraction from mouse stool samples was carried out using the Quick-DNA Fecal/Soil Microbe 96 Kit (Zymo Research, CA, USA) following the manufacturer’s protocols. The hypervariable region V4 of the bacterial 16S rRNA gene, 515F (5′-GTGYCAGCMGCCGCGGTAA-3′) and 806R (5′-GGACTACNVGGGTWTCTAAT-3) was amplified and sequenced on the Illumina MiSeq platform (2 × 250 bp) (Illumina, CA, USA). The raw sequencing reads were processed via the DADA2 V1.16 (RRID:SCR_023519) data processing pipeline to generate amplicon sequence variants (ASVs). Taxonomic assignments were made using the Silva database V138.1 (RRID:SCR_006423), with a classification confidence threshold set at *p* < 0.5 for assigning unclassified taxa at their respective taxonomical levels. PCR negative controls and extraction controls for DNA extraction and sequencing were included in the analysis. Notably, all negative controls yielded fewer than 500 reads, indicating that background noise minimally impacted the data analysis. Sequencing reads from all the samples were rarified to 10,000 reads per sample. Further, ASVs were used for functional inference using PICRUSt2 [38]. The resultant Kyoto Encyclopedia of Genes and Genomes Orthologs (KOs) were used for gut-brain modules (GBM) detection [39]. GBM analysis was performed using the R version of the Gomixer tool and a non-parametric Wilcoxon test was used to compare NaCl and sodium valerate groups as GBM measures were not normally distributed. All of the microbiome analysis was done in R.

### General statistical approaches

General statistical analysis (non-microbiome and non-RNAseq analysis) was conducted using GraphPad Prism 8 (GraphPad, CA, USA, RRID:SCR_002798). For the calculation of ethanol consumption during the DID procedure, it was expressed as grams of ethanol per kilogram of body weight (g/kg body weight), where 20% ethanol intake was determined as follows: multiplying the drinking volume by the ethanol percentage within that volume and the density of ethanol, and then dividing this by the mouse’s body weight in kilograms. The normality of data distributions was tested by the Shapiro–Wilk test and by Q-Q plots. Levene’s test was used to test the equality of variances across groups. Unpaired Student’s *t*-tests were used to compare behavioral testing, GABA levels, and histone acetylation measurements between the sodium valerate and NaCl groups. To evaluate the impact of antibiotic cocktail administration on ethanol consumption and BEC between the groups receiving Abx and PBS at baseline and after treatment, a mixed ANOVA test was employed, given repeated measures from the same mouse from the two-time points. Similarly, mixed ANOVA tests were applied to compare various SCFA levels before and after Abx administration. Analysis of variance (ANOVA) was used for comparisons involving more than two groups when there are equal variances across samples. For instance, ordinary one-way ANOVA was employed to assess the impact of all SCFA supplementation on ethanol consumption and BEC. Corrections for multiple comparisons in ANOVA were made using Tukey’s post-hoc test.

The food intake was assessed by providing a pre-weighed amount of food in their cage hopper and then weighing the remaining food inside the cage and in the hopper daily. To assess daily fluid intake, the sodium valerate and NaCl bottles were pre-weighed, and the remaining amount was deducted after the measurement period. Food and fluid intakes were normalized to mouse body weight by dividing the average intake by the average body weight.

For all the above analyses, the *p* values were reported with the respective *F*- or *T*-statistic and associated degrees of freedom (df).

## Results

### Antibiotic administration increases ethanol consumption but reduces fecal SCFA levels

**Fig. 1:**
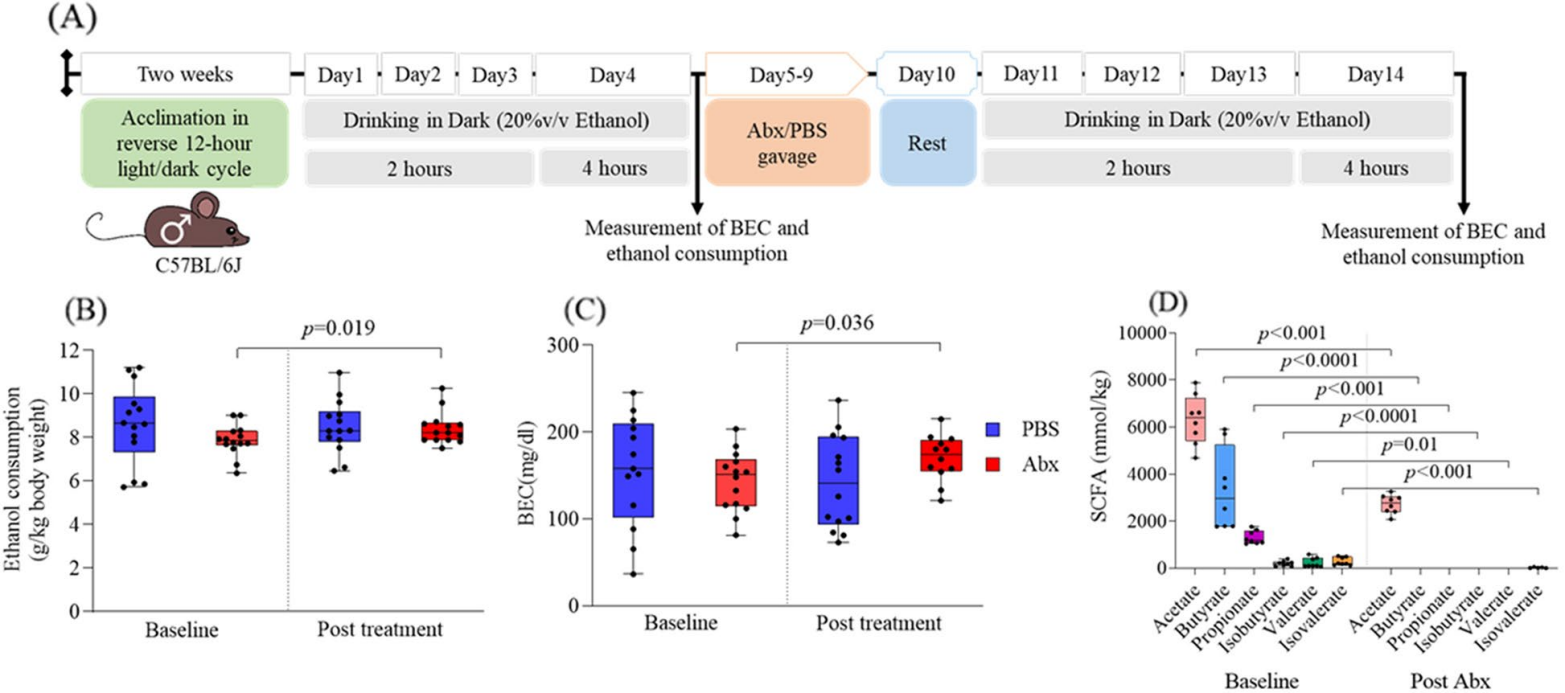
Effect of oral antibiotics on ethanol consumption, BEC, and levels of SCFA in stool. **A** A schematic depiction illustrating the DID paradigm and Abx treatment. The light gray horizontal bar represents the 20% v/v ethanol drinking for 2 h during the first 3 days and for 4 h on the fourth day. The light orange horizontal bar symbolizes the administration of antibiotics (Abx) or PBS by oral gavage. The light blue horizontal bar represents the rest day post-antibiotic treatment. The black arrow represents the measurement of ethanol consumption and BEC at the completion of the 4 h of ethanol drinking. **B** Ethanol consumption normalized by body weight at the end of the 4 h drinking period (*n* = 14 mice/group). **C** BEC at the end of the 4 h drinking period (*n* = 14 mice/group). **D** Effect of Abx administration on SCFA levels in the stool (*n* = 7 mice). In **B** and **D** of the figure, a mixed ANOVA was used to compare the two groups. The data in all three panels (**B**–**D**) are presented using box plots. The significant *p* values are presented within the figure

We measured ethanol consumption (unit: g/kg body weight) and BEC (unit: mg/dl) in adult male mice (*n* = 14 mice/group) using a DID paradigm at baseline and after Abx treatment. A schematic diagram illustrating the administration of Abx and the DID procedure is depicted in Fig. 1A. At baseline, no statistically significant differences in ethanol consumption (Fig. 1B) or BEC were noted (Fig. 1C). Following a 10-day treatment period with Abx, we once again employed the DID paradigm in both the Abx-treated mice and PBS control mice. Notably, after this treatment period, the Abx-treated mice exhibited a significantly higher level (*p* = 0.03, *t* = 2.44, df = 11; Fig. 1B) of ethanol consumption and BEC (*p* = 0.03, *t* = 2.47, df = 11; Fig. 1C) compared to their baseline levels. In contrast, the PBS control mice did not display a significant difference in either consumption or BEC when compared to their baseline levels.

To test whether the levels of SCFAs are altered in Abx-treated mice, we analyzed the SCFA at baseline and after Abx treatment in the mice’s stool. At baseline, SCFAs such as acetate, butyrate, and propionate are usually found at high concentrations, while isobutyrate, valerate, and isovalerate are found at lower concentrations (*n* = 7 mice). All SCFAs were significantly abolished after antibiotic treatment (*p* < 0.05), with only minimal levels of acetate (mean ± SD = 2722.21 ± 372.26 mmol/kg stool) and isovalerate (mean ± SD = 23.8 ± 17.02 mmol/kg stool) remaining (Fig. 1D).

### Sodium valerate supplementation reduces ethanol consumption, BECs, and anxiogenic behavior

**Fig. 2:**
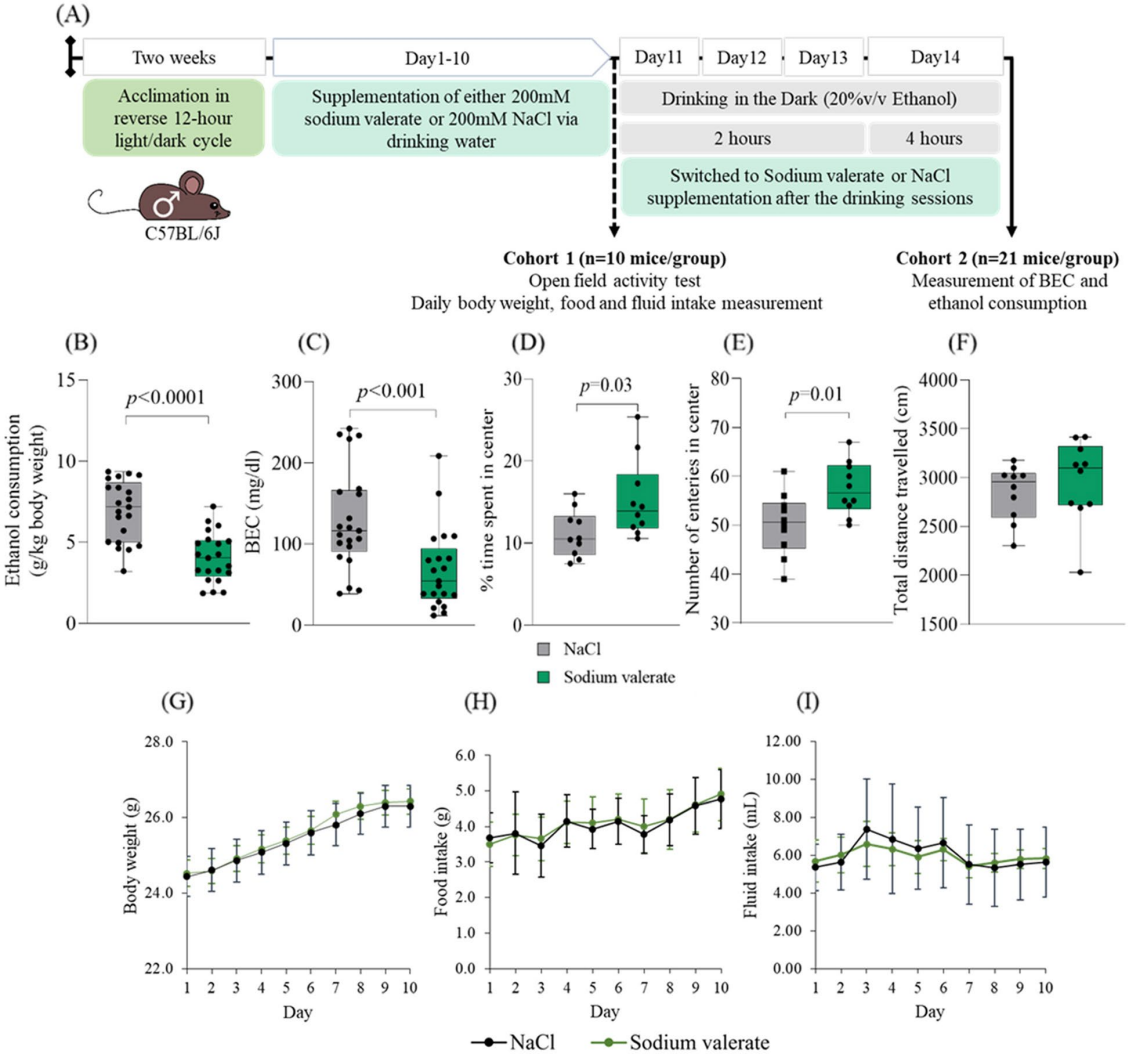
Effect of sodium valerate on ethanol consumption, BEC, anxiety, and diet. **A** This schematic illustrates the incorporation of sodium valerate and NaCl supplementation into the DID paradigm. The light gray horizontal bar represents the 20% v/v ethanol drinking for 2 h during the first 3 days and for 4 h on the fourth day within the DID paradigm. The light green horizontal bar represents either sodium valerate in sodium valerate-supplemented mice or NaCl supplementation in control mice. This supplementation continued throughout the entire experiment, except during DID sessions. The dashed black arrow indicates open field activity testing after completing 10 days of supplementation with sodium valerate or NaCl in cohort 1. Throughout this period, mice in cohort 1 were also monitored for daily changes in body weight, food intake, and fluid intake. Additionally, the solid black arrow indicates the measurement of ethanol consumption and BEC through blood collection after the completion of the 4 h ethanol drinking session in cohort 2. **B** Ethanol consumption, and **C** BEC in mice supplemented either with sodium valerate or NaCl (*n* = 21 mice/group). **D** Percentage of time spent in the center, **E** number of center entries, and **F** distance traveled in the center area during the open field test of mice supplemented with sodium valerate or NaCl (*n* = 10 mice/group). In **B**–**F**, a Student *t*-test was employed to compare groups. In each of the four panels (**B**–**F**), the data are depicted using boxplots. **G** Daily body weight, **H** food consumption, and I intake of sodium valerate and NaCl in respective groups over a 10-day period. Each line represents mean ± SD for each group. The significant *p* values are presented in the figure

We next investigated the effect of individual SCFAs (sodium butyrate, sodium acetate, and sodium valerate) supplementation (daily for 10 days in drinking water) on ethanol consumption in mice (*n* = 7 mice/group) via the DID paradigm. Notably, sodium valerate-supplemented mice consumed less ethanol than both NaCl and other SCFA-supplemented mice. A significant difference (*p* = 0.004, *f* = 5.04, and df = 24) was observed between sodium valerate and sodium butyrate-supplemented mice (Supplementary Figure S1A, B). We repeated the sodium valerate supplementation study in a larger cohort of male mice (*n* = 21 mice/group) (Fig. 2A). The effect of sodium valerate supplementation on reducing ethanol consumption (*p* < 0.0001, *t* = 4.09, and df = 54; Fig. 2B) and BEC (*p* < 0.001, *t* = 3.53, and df = 40; Fig. 2C) was significant. Sodium valerate supplementation led to a 40% reduction in ethanol consumption, with a median of 4.03 g/kg body weight compared to 7.16 g/kg consumed body weight in the NaCl-supplemented mice. Additionally, BEC was lowered by 53% in sodium valerate-supplemented mice, with a median of 54.3 mg/dl compared to a median of 116.3 mg/dl in NaCl-supplemented control mice.

We examined the effects of sodium valerate and NaCl on anxiety-like behavior (*n* = 10 mice/group) after 10 days of supplementation by an open-field activity test. As shown in Fig. 2D, E, sodium valerate supplementation produced a significant increase in the percentage of time spent in the center are [mean ± SD = 15.29 ± 4.82; *p* = 0.03, *t* = 2.36 and df = 18] and the number of entries in the center [mean ± SD = 57.50 ± 5.48; *p* = 0.01, *t* = 2.73, and df = 18] during a 10-min exploration period. However, it did not lead to a significant change in the distance traveled (Fig. 2F). Overall, supplementation of sodium valerate reduces anxiety-like or approach-avoidance behavior in mice. Although the mice gained weight throughout the study, the average weight did not differ significantly (*n* = 10 mice/group) between the sodium valerate and NaCl groups (Fig. 2G). In addition, we observed no statistical difference (*n* = 14 mice/group) in food consumption or intake of sodium valerate and NaCl among the respective groups (Fig. 2H, I).

### Enhanced GABA levels following sodium valerate supplementation

**Fig. 3:**
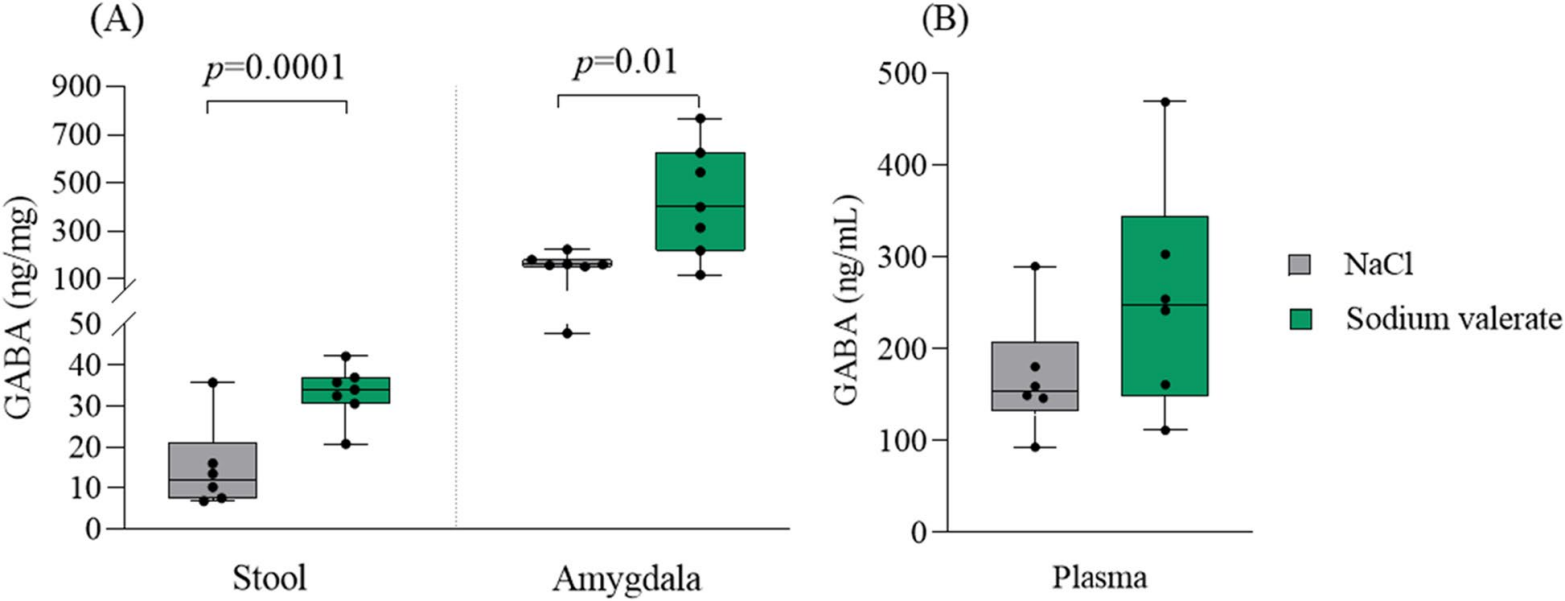
Effect of sodium valerate supplementation on GABA levels. **A** The levels of GABA were measured in stool and amygdala tissues (*n* = 7 mice/group), and **B** plasma samples (*n* = 6 mice/group) of sodium valerate and NaCl control mice after 10 days of supplementation following the DID paradigm. The data are illustrated with boxplots. Student *t*-test was employed to compare groups. The significant *p*-values are provided in the figure

We next measured the levels of GABA in the amygdala, stool, and blood of the mice that underwent the DID paradigm, comparing those supplemented with sodium valerate to those supplemented with NaCl. Our findings revealed that sodium valerate supplementation led to elevated GABA levels in the stool (*p* = 0.0001, *t* = 5.71, df = 11) and the amygdala (*p* = 0.01, *t* = 2.78, df = 12), compared to the control group supplemented with NaCl (Fig. 3A). A similar trend was also evident in plasma (Fig. 3B) but was not significant. No detectable levels were observed when directly testing valeric acid with the ELISA assay.

### Sodium valerate supplementation increases histone acetylation in the amygdala

**Fig. 4:**
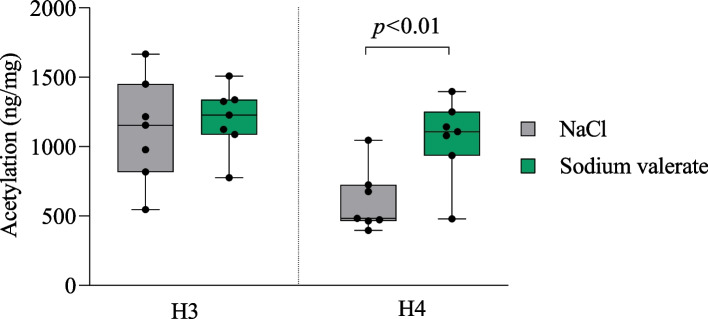
Effect of sodium valerate supplementation on levels of histone acetylation. Acetylation of H3 and H4 in the amygdala of sodium valerate and NaCl control mice after 10 days of supplementation following the DID paradigm (*n* = 7 mice/group). The data are illustrated with boxplots. Student *t*-test was employed to compare groups. The significant *p* value is mentioned within the figure

We quantified the total acetylation levels of histone H3 and H4 in the amygdala of mice subjected to the DID paradigm following supplementation with sodium valerate or NaCl (*n* = 7 mice/group). We found H4 acetylation significantly increased in mice supplemented with sodium valerate compared to NaCl control (*p* < 0.01, *t* = 3.20, df = 12), while the observed increase in H3 acetylation with sodium valerate was not statistically significant in the amygdala of these mice (Fig. 4).

### Amygdala transcriptome analysis in mice supplemented with sodium valerate

**Fig. 5:**
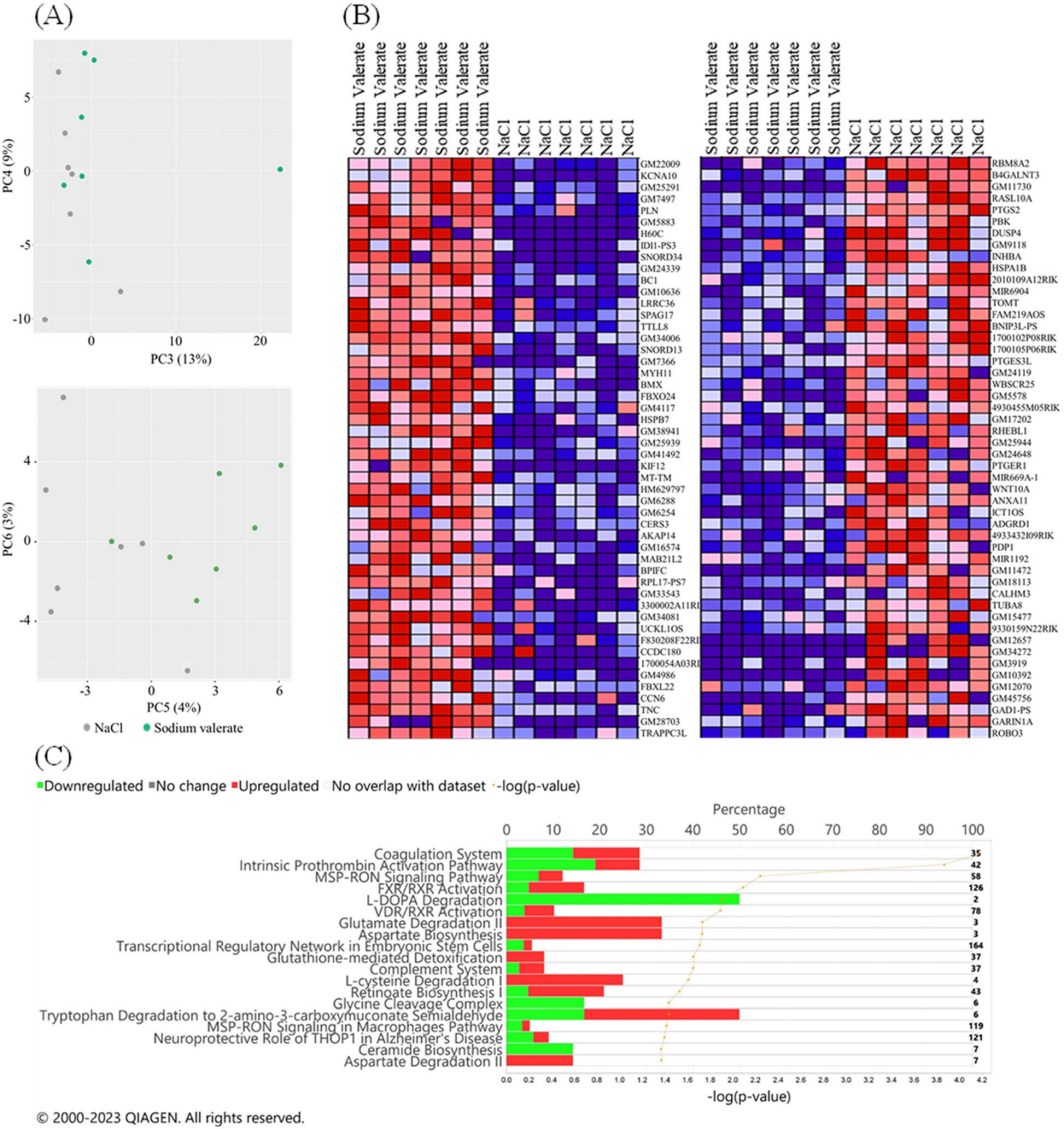
Effect of sodium valerate on gene expression in the amygdala. **A** PCA plot illustrates sample characteristics from both sodium valerate and NaCl groups (*n* = 7 mice/group) based on gene expression levels, with each dot representing a sample. **B** The heatmap displays the top 50 upregulated and top 50 downregulated DEGs in mice supplemented with sodium valerate (left column) and NaCl (right column). The expression values are depicted as ranging from red (high expression) to pink (moderate), light blue (low), and dark blue (lowest expression). **C** Highlights some of the top canonical pathways identified by IPA that are affected by sodium valerate supplementation. The representation of pathway regulation is expressed as a percentage (top axis) using bar graphs, with respective *p* values (bottom axis) derived from right-tailed Fisher’s exact test represented by the yellow line

To gain a molecular understanding of the effects of sodium valerate supplementation on brain function, we performed bulk RNA sequencing (RNA-seq) analysis on the amygdala tissue of mice that underwent the DID paradigm after sodium valerate or NaCl (*n* = 7 mice/group) supplementation. Principal component analysis (PCA) showed no clear distinction between sodium valerate-supplemented mice and NaCl-supplemented mice at PC1 and PC2 components, but a clear separation in PC3 to PC6 (Fig. 5A), suggesting sodium valerate supplementation has a marked influence on gene expression in the amygdala. DESeq2 identified a total of 301 DEG at the adjusted *p* value < 0.25, 126 genes at the adjusted *p* value < 0.10, and 75 genes at the adjusted *p* value < 0.05. Among the 75 significant DEG, 47 genes showed upregulation, while 28 displayed downregulation. The heatmap displays the top 50 genes in the sodium valerate versus NaCl control (Fig. 5B). Among these genes, the top 5 protein-coding genes with the highest expression in sodium valerate-supplemented mice are *Kcna10* (potassium voltage-gated channel, shaker-related subfamily, member 10), *Pln* (phospholamban), *H60C* (histocompatibility antigen 60c), *Idi1* (isopentenyl-diphosphate delta isomerase, pseudogene 3) and *Snord34* (small nucleolar RNA, C/D box 34). The top five most strongly down-regulated genes in the sodium valerate group are *Rbm8a2* (RNA binding motif protein 8A2), *B4galnt3* (beta-1,4-N-acetyl-galactosaminyl transferase 3), *Rasl10a* (RAS Like Family 10 Member A), *Ptgs2* (prostaglandin-endoperoxide synthase 2), and *Pbk* (PDZ binding kinase). *PTGS2* is a prostanoid producer that responds to inflammation. Its downregulation in the sodium valerate-supplemented group (log_2_Fold change: − 0.65; adjusted *p* = 0.0002) indicates a potential anti-inflammatory effect of valerate. Mitogen-activated protein kinases (MAPKs), a group of protein kinases that regulate critical inflammatory processes are also downregulated at the RNA level in the sodium valerate supplemented group. As SCFAs may function in the brain through G protein-coupled receptors (GPCRs), we examined gene expression levels of 85 GPRs in the RNAseq data. We found that *GPR56* and *GPR158* are highly expressed among all these GPCRs. *Gpr56*, whose expression has been associated with antidepressant response, was upregulated in sodium valerate-supplemented mice (log_2_Fold change: 0.28; unadjusted *p* = 0 < 0.01). Conversely, *GPR158* is associated with depression after chronic stress and is downregulated in the sodium valerate-supplemented group (log_2_Fold change − 0.26; unadjusted *p* < 0.01). Notably, we found that six mitochondrial genes encoding subunits of the enzyme NADH dehydrogenase (Complex I) were consistently upregulated in the sodium valerate-supplemented group. IPA was performed to identify changes to the molecular signaling pathways and to comparatively assess the sodium valerate effects in the amygdala. The regulated pathways predicted in sodium valerate-supplemented mice are associated with the downregulation of L-dopa degradation, upregulation of aspartate biosynthesis, glutamate degradation, tryptophane degradation, the bidirectional regulation of pulmonary blood coagulation, retinoate biosynthesis, complement system, FXR/RXR activation, LXR/RXR activation, as well as the upregulation of L-cysteine degradation and the downregulation of glycine cleavage complex ceramide biosynthesis, etc. (Fig. 5C). Thus, sodium valerate treatment induces significant changes in gene expression spanning various signaling processes including neuroinflammation, neurotransmission, mitochondria regulation, and GPCR signaling.

### Sodium valerate supplementation shifts the gut microbiota community and gut-brain modules

**Fig. 6:**
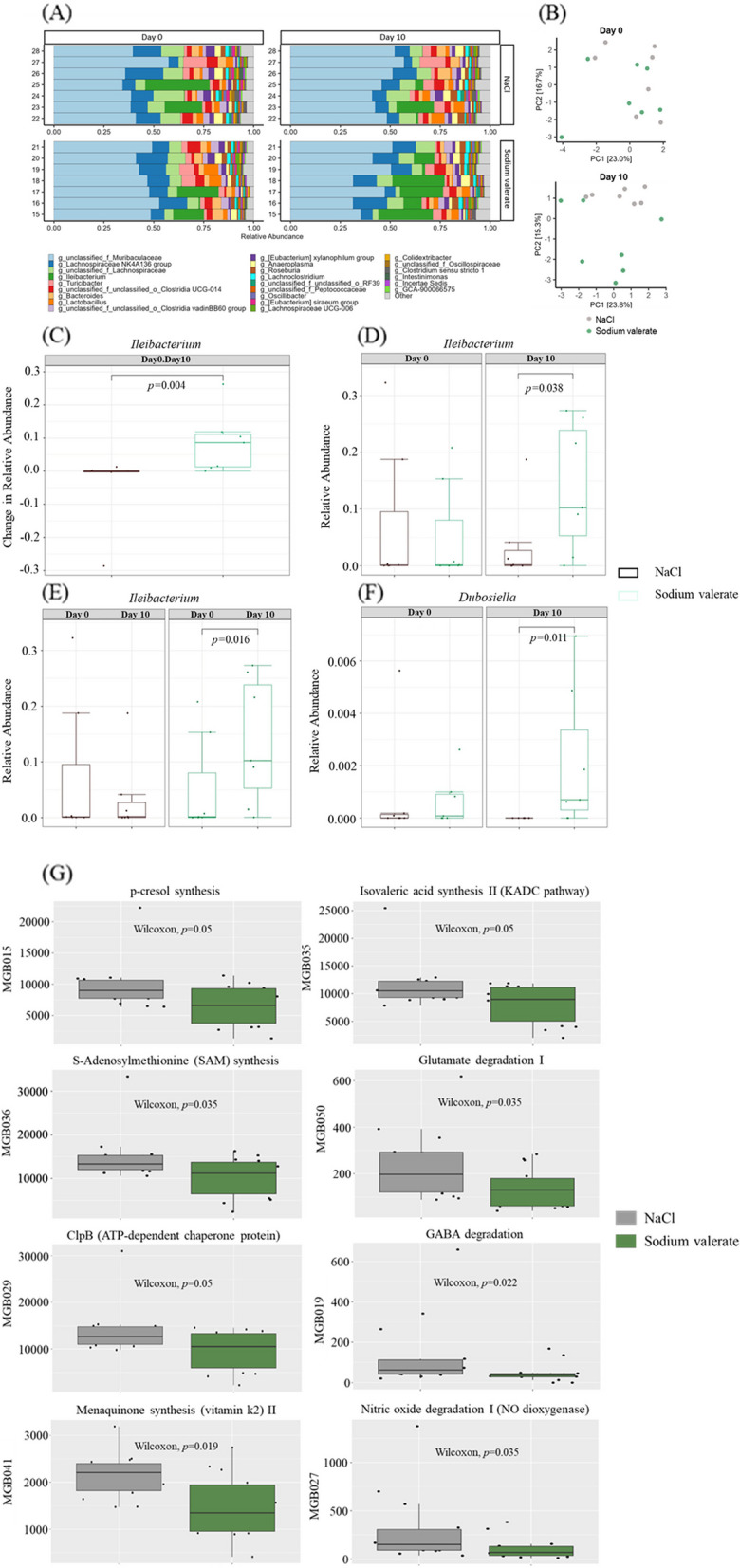
Gut microbiome, gut metabolites, and gut-brain modules in sodium valerate-supplemented mice. **A** Stacked bar plot illustrates the percentage of the top 25 genera present in stool samples from sodium valerate and NaCl-supplemented mice (*n* = 7 mice/group) as identified by sequencing of V4 16S amplicons. **B** PCA plots showcase the variation in beta diversity between mice supplemented with NaCl and sodium valerate at day 0 and day 10. Each dot represents a sample from an individual mouse. **C** Displays of the changes of relative abundance of *Ileibacterium* after the 10-day period of sodium valerate and NaCl supplementation from the baseline day 0. **D** The differences in relative abundance of *Ileibacterium* between the groups at day 0 and day 10. **E** Highlights of the variations in the relative abundance of *Ileibacterium* within each group at day 0 and day 10. **F** Demonstration of the differences in the relative abundance of *Dubosiella* between the groups at day 0 and day 10. In **C**–**G**, the data are presented using box plots. **G** Depiction of predicted gut-brain modules in mice supplemented with sodium valerate and NaCl. Relative abundance difference of specific taxa was identified using the Wilcoxon-sum rank test. Here *n* = 7 mice/group were used for gene expression analysis

We performed 16S rRNA gene sequencing of stool samples collected before and after sodium valerate treatment (*n* = 7 mice/group). The most abundant genera were unclassified *Muribaculaceae*, *Lachnospiraceae* NK4A136 group, and unclassified *Lachnospiraceae* (Fig. 6A). Permutational multivariate analysis of variance (PERMANOVA) tests were performed on CLR-transformed data to test for beta-diversity differences between sodium valerate and NaCl-supplemented groups (*n* = 14 mice/group). Prior to any supplementation at day 0, no significant difference was observed between the groups. However, after 10 days of supplementation, the groups showed increased dissimilarity and exhibited distinct clustering patterns when visualized in a PCA plot, indicating a supplementation-related impact on the composition of the gut microbiome (Fig. 6B). Among all the genera analyzed, an increase in abundance was noted only in the *Ileibacterium* genera in sodium valerate supplemented mice (adjusted *p* = 0.24, unadjusted *p* = 0.004) (Fig. 6C). This finding was further confirmed via Wilcox tests on the relative abundance at day 10 between sodium valerate and NaCl mice (unadjusted *p* = 0.038) (Fig. 6D), and the difference in abundance between day 0 and day 10 for the sodium valerate mice (unadjusted *p* = 0.011), which supported an increase in *Ileibacterium* in sodium valerate supplemented mice (Fig. 6E). Further analysis with DESeq2 found that the genus *Dubosiella* was significantly (log_2_Fold change = 7.84, adjusted *p* = 9.46E − 5) more abundant in mice after 10 days of sodium valerate supplementation (Fig. 6F). This finding was confirmed with a Wilcox test (unadjusted *p* = 0.011).

To determine the neuroactive potential of the valerate-altered gut microbiota, we inferred neuroactive compound production and degradation process based on gut-brain module (GBM) analysis. Thirty-four GBMs were identified in the collected samples, and 8 GBMs exhibited significant differences (*p* < 0.05) between the sodium valerate and NaCl-supplemented mice. Interestingly, all 8 GBMs showed a significant decrease in valerate-treated mice (Fig. 6G), including p-Cresol synthesis, Isovaleric acid synthesis II, S-Adenosylmethionine synthesis, Glutamate degradation I, GABA degradation, Nitric oxide degradation I, ClpB (ATP-dependent chaperone protein), and Menaquinone synthesis (vitamin K2) II.

## Discussion

Our study discovered that a sodium salt of gut microbial metabolite valeric acid reduces binge-like alcohol consumption in mice. This effect is associated with an increase of GABA levels in the periphery and brain, modulation of brain epigenetics and transcriptomics, and an impact on the gut microbiome composition.

Previous research has demonstrated the involvement of gut microbiota in alcohol consumption, [40–42] and the disruption of gut microbiota through antibiotics has been shown to increase ethanol consumption in mice [43]. In agreement with this study, we found that Abx treatment significantly increased voluntary ethanol consumption levels in a binge-like ethanol-drinking paradigm in mice. However, the opposite effect was reported in Wistar-derived high-drinker UChB rats [44], it is worth noting that it utilized an ad libitum ethanol access paradigm leading to lower BEC. In addition, a different antibiotics regimen, and animals were also used in their study. Regardless, our work reveals an important relationship between gut microbiota and ethanol consumption behavior and supports the use of microbial-targeted approaches to study gut-brain interactions in alcohol-drinking behavior. In our mouse model, the production of intestinal SCFAs, including acetate, butyrate, isobutyrate, propionate, valerate, and isovalerate, was significantly suppressed by the Abx treatment. This finding is consistent with previous reports that have implicated SCFA levels in the modulation of alcohol consumption in mice [24, 26]. Additionally, it is well-established that oral antibiotic treatment can effectively suppress the levels of intestinal SCFA [45, 46].

When provided various SCFAs to mice as supplements, we observed no statistical changes in alcohol intake when sodium acetate and butyrate were supplemented. Prior research suggests that acetate might encourage heavy drinking, providing a reward in the form of added energy from calories or influencing adenosinergic adaptation mechanisms [26]. Studies have shown that sodium butyrate does not influence alcohol self-administration in non-dependent rodents but may reduce drinking in alcohol-dependent or antibiotic-treated rodents [47, 48]. Interestingly, when we supplemented valeric acid, we observed a significant reduction in ethanol consumption and BEC. According to reports, BEC in humans ranging from 50 to 70 mg/dl may result in mild impairment in motor skills [49]. In our study, mice treated with sodium valerate exhibited a BEC of approximately 54.3 mg/dl, which is relatively low. However, whether these low levels are associated with any impairment requires further evaluation through behavioral assessments post-drinking in the DID paradigm.

Valeric acid also presents naturally in various plant sources, including *Valeriana wallichii* and *Valeriana officinalis*, etc. One study has shown that *Valeriana wallichii* extract reduces chronic ethanol intake in animal models. However, *Valeriana wallichii* extract contains a variety of active constituents. The exact compound that is responsible for reduced ethanol intake has not been studied. In our study, sodium valerate supplementation did not affect body weight, food intake, or fluid drinking. This suggests that the observed reduction in alcohol consumption was not due to changes in fluid or weight regulation. A recent study reported a decrease in fecal isovalerate (an isomer of valeric acid) linked to increased alcohol drinking in humans [50], which further reiterates the potential of valeric acid in regulating ethanol consumption.

The molecular mechanisms that underlie alcohol-drinking behaviors are intricate and multifaceted [51]. Anxiety can promote alcohol-drinking behaviors in both humans and animals, and excessive drinking increases anxiety-like behavior [50, 52, 53]. Our study suggests that sodium valerate supplementation has potential anxiolytic effects in mice. Interestingly, *Valeriana wallichii* and *Valeriana officinalis*, plant reservoirs of valeric acid and other compounds, have been used as supplements to address insomnia and anxiety due to their sedative attributes [54–56]. GABA may play a role in reducing depression and anxiety linked to alcohol dependence, as lower GABA levels are associated with these conditions [57, 58]. In our study, sodium valerate supplements led to increased GABA levels in stool and the amygdala. Valproic acid, a structural analog of valeric acid, is a popular antiepileptic drug with GABAergic activity. Some research suggests that valproic acid may raise GABA levels in the brain by inactivating α-ketoglutarate dehydrogenase involved in the breakdown of GABA [59]. Whether valeric acid acts in a similar fashion warrants further investigation.

Further, increased levels of GABA detected in stool samples from mice supplemented with sodium valerate suggest that the gut microbiome may be involved in GABA regulation. Previous studies have identified a variety of GABA-producers and degraders in the gut microbiome [60, 61]. Indeed, our gut-brain module analysis revealed a decrease in GABA degradation by the gut microbiome in sodium valerate-supplemented mice. It will be interesting to examine the ability of GABA modulation by *Ileibacterium* and *Dubosiella*, two bacterial genera that are significantly increased during sodium valerate supplementation. However, it is also possible that changes in *Ileibacterium* and *Dubosiella* abundance are responses to administered sodium valerate or increased GABA. A report indicated that the metabolic disorder induced by chronic alcohol consumption caused a decrease in the relative abundance of *Ileibacterium-* [62]. Under physiological conditions, it has been widely believed that GABA does not cross blood blood–brain barrier [63, 64], whereas SCFAs have the ability of brain penetration [65]. The impact of gut-derived GABA on brain function and drinking behavior, therefore, warrants further investigation. It is also possible that valeric acid can directly cross blood blood–brain barrier and regulate GABA levels in the brain or act indirectly through the gut-brain axis. These intriguing observations suggest a potential connection between valeric acid supplementation, heightened GABA levels, and a reduction in alcohol consumption.

There is consistent evidence that acute and chronic alcohol exposure modulate histone acetylation in the amygdaloid circuitry, leading to alcohol tolerance and dependence [66, 67]. Our study revealed increased acetylation of histone H4 in the amygdala of sodium valerate-supplemented mice. Previous findings confirm that intermittent alcohol exposure decreased histone acetylation in the amygdala, which may be related to the ethanol-induced increase in histone deacetylase (HDAC) [68]. Similarly, the administration of HDAC inhibitors like sodium butyrate increases histone acetylation and suppresses anxiety or depression-like behaviors in mice. Our results suggest that HDAC inhibitors such as sodium valerate may be able to reverse the effects of ethanol via HDAC-induced epigenetic changes in the amygdala, consequently resulting in reduced ethanol consumption.

Increased histone acetylation leads to a more open structure of chromosomes, thus promoting gene transcription. Our data suggests that another potential mechanism by which valeric acid attenuates alcohol drinking is through its effects on transcriptional regulation in the brain. A downregulation of inflammatory molecules such as *Ptgs2* and *MAPK* was identified in valerate-treated mice. The immune modulatory effect of valerate has been shown in experimental mouse models of colitis and multiple sclerosis, mediated by suppressing Th_17_ cells and the enhancement of IL-10 production [69]. Numerous studies have demonstrated that SCFAs modulate transcription of a wide range of genes associated with behaviors [23]. SCFAs are known to regulate GPCRs such as *GPR41*, *GPR43*, and *GPR109A*, all of which are critical in regulating neuroinflammation, depression, and anxiety-like behaviors [70]. Our bulk RNAseq analysis showed upregulation of *GPR56* and downregulation of *GPR158* in the amygdala region of the brain in valerate-treated mice. *GPR158* is a novel regulator of stress-responsive behaviors and is highly upregulated in people with major depression disorder [71]. By contrast, *GPR 56* activation has an antidepressant effect [72]. Valerate acid may regulate two GPRs of opposing effects to control anxiety or depression behavior, thus indirectly influencing moderating drinking behavior.

Our study has some limitations. All experiments in the current study were performed in male mice, further investigation is necessary to ascertain if there also exists an association between sodium valerate and alcohol intake in females. The study was tested on alcohol-independent mice. Future studies will be conducted to assess the treatment effects of sodium valerate supplementation on alcohol-dependent animals. Further research is needed to fully understand the underlying mechanisms of action of valeric acid on voluntary alcohol drinking.

## Conclusion

Sodium valerate supplementation shows promise as a novel intervention to reduce alcohol consumption. This effect potentially acts through the modulation of multiple molecular targets associated with the pathogenesis of excessive alcohol use. Our study contributes to the growing understanding of the gut-brain axis and provides insights into potential therapeutic strategies for excessive alcohol consumption and related anxiety.

### Supplementary Information

Supplementary Material 1: Supplementary Figure 1. SCFA supplementation effect on ethanol consumption. (A) Ethanol consumption and (B) BEC in mice supplemented with SCFAs and NaCl (n=7 mice/group). The data is depicted using boxplots. One way ANOVA was employed, followed by Tukey’s post-hoc analysis to compare ethanol consumption and BEC levels between groups. The significant *p*-value is displayed within the figure.

## Data Availability

The raw sequencing data can be accessed in the Sequence Read Archive (SRA) database under the accession number PRJNA1034104.

## Abbreviations

Abx: Antibiotic cocktail
BEC: Blood ethanol concentration
DEG: Differentially expressed genes
DeSeq2: Differential expression analysis of RNA-Seq 2
DID: Drinking in the dark
GABA: γ-Aminobutyric acid
GBM: Gut brain module
GPCR: G protein-coupled receptors
HDAC: Histone deacetylase
IPA: Ingenuity pathway analysis
NaCl: Sodium chloride
MAPK: Mitogen-activated protein kinases
PBS: Phosphate-buffered saline
PCA: Principal component analysis
SCFA: Short-chain fatty acid
