# Supplementary material for: Short-chain fatty acid valerate reduces voluntary alcohol intake in male mice
Source: Microbiome. 2024 Jun 17;12:108. doi: 10.1186/s40168-024-01829-6 (PMC11181657; doi:10.1186/s40168-024-01829-6)
Supplement: Supplementary file 2 — Supplementary Material 1: Supplementary Figure 1. SCFA supplementation effect on ethanol consumption. (A) Ethanol consumption and (B) BEC in mice supplemented with SCFAs and NaCl (n=7 mice/group). The data is depicted using boxplots. One way ANOVA was employed, followed by Tukey’s post-hoc analysis to compare ethanol consumption and BEC levels between groups. The significant p-value is displayed within the figure. [file 40168_2024_1829_MOESM1_ESM.docx]

**Supplementary figure: SCFA supplementation effect on ethanol consumption**

**
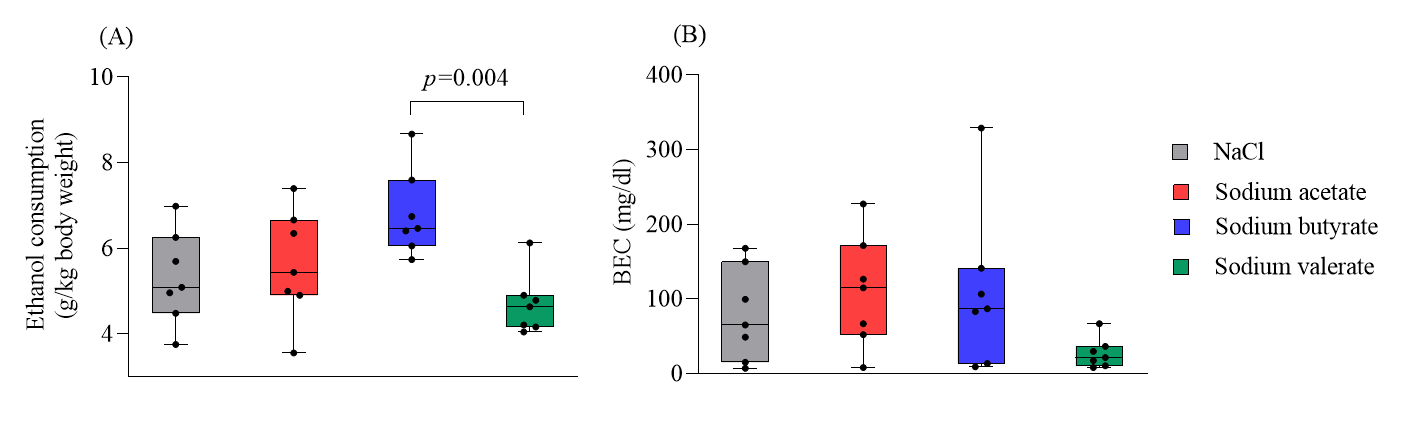
**(A) Ethanol consumption and (B) BEC in mice supplemented with SCFAs and NaCl (n=7 mice/group). The data is depicted using boxplots. One way ANOVA was employed, followed by Tukey’s post-hoc analysis to compare ethanol consumption and BEC levels between groups. The significant *p*-value is displayed within the figure.
